# Study on the optimization of silicone copolymer synthesis and the evaluation of its thickening performance

**DOI:** 10.1039/c7ra13645e

**Published:** 2018-02-26

**Authors:** Qiang Li, Yanling Wang, Qingchao Li, Gomado Foster, Chuang Lei

**Affiliations:** College of Petroleum Engineering, China University of Petroleum (East China) Qingdao 266580 China wangyl@upc.edu.cn; PetroChina Huabei Oilfield Company Renqiu 062500 China

## Abstract

Silicone polymer shows high performance for thickening supercritical carbon dioxide and has become a well-known target because it is inexpensive and environmentally friendly. In this study, siloxane polymer was synthesized by a copolymerization reaction. The synthesis conditions of the silicone polymer were optimized using a Box–Behnken design, and the yield from the process was considered as an evaluation criterion in the screening of the synthesis process. The thickening effect of the polymer was evaluated using an in-house-built ball viscometer with operation pressure not exceeding 30 MPa. The experiments clearly showed that temperature is the most crucial factor for the synthesis process. At higher preparation temperatures (>90 °C), the yield significantly decreased from the process. The stability of the yield was influenced by the change in the molar ratio and amount of the catalyst used in the preparation. The most optimal preparation parameter for the synthesis was at a temperature of 90 °C, with an aminopropyltriethoxysilane-to-methyl triethoxysilane molar ratio of 2 : 1, and 0.09 g of tetramethylammonium hydroxide as a catalyst. The test yield (84.51%) coordinated well with the predicted yield of 83.72%. Adding 3 wt% siloxane to pure carbon dioxide thickened it 5.7 times at 35 °C and 12 MPa. An enhanced yield trend was observed with increasing pressure and a temperature range of 35–55 °C. The application of CO_2_ fracturing technology can help to reduce the greenhouse effect and the environmental pollution caused by fluoropolymers as thickeners when silicone polymer is deployed as a thickener for CO_2_.

## Abstract 2

The synthesis process of the silicone ternary copolymer.

## Graphical abstract

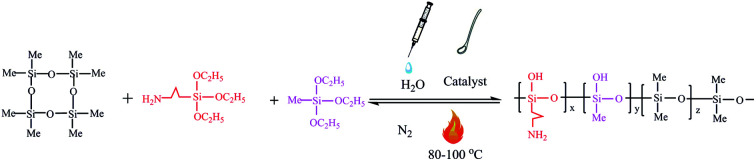

## 1. Introduction

Hydraulic fluid is widely used to enhance oil or gas recovery in the fracturing process.^1,2^ As thickeners and proppants, cellulose-based compounds are used to improve the viscosity of hydraulic fracturing fluid and brace behavior conferred to the cracks in the fracturing process.^3^ However, with the appearance of disadvantages, including large water consumption, water pollution, stratum pollution, and a low flowback rate, the hydraulic fracturing process is gradually being replaced by other fracturing processes.^4–6^ Supercritical CO_2_ (Sc-CO_2_) has proven to be a good alternative for enhanced oil recovery and also fracturing fluid formulation due to its environmental friendliness and low cost.^7–10^ However, it should be noted that cellulose is used only as a proppant in CO_2_ fracturing technology due to the demand of brace behavior to cracks, and therefore, the mobility control of Sc-CO_2_ has become one of the principal challenges encountered in oil and gas field development engineering.^11^ This problem is mitigated by thickening the Sc-CO_2_ using silicone polymers that dissolve in many organic solvents such as toluene and cyclohexane. The viscosity of a mixture of silicone polymer, co-solvent, and Sc-CO_2_ has been previously measured, and the data verify that a series of silicone polymers are effective in thickening Sc-CO_2_.^12,13^ This has generated interest in the development of linear siloxane polymers for use as silicone thickeners.^14^

Currently, fluoropolymers have been recognized and deployed as good alternatives due to their enhanced solubility in CO_2_, and they have been applied in a wide range of CO_2_ fracturing projects in various oilfields.^37–41^ Despite their enhanced solubility, the application of fluoropolymers is limited because they tend to pollute the groundwater during the oilfield development process and they are very expensive when deployed as a thickener. Because of this, much research has been carried out to improve the performance of siloxane polymer with the assistance of a cosolvent.^15,16^ Because the performance of siloxane polymers is limited by their low degree of thickening, a large amount of cosolvent is required to achieve a higher viscosity necessary for thickening supercritical CO_2_. In view of the above limitation, reducing the amount of cosolvent and improving the thickening ability has become a challenge. From previous studies performed on siloxane polymers, it is believed that the molecule of siloxane polymer, which thickens supercritical CO_2_, should contain CO_2_-philic groups and CO_2_-phobic groups in appropriate proportions. More specifically, the main chain of siloxane polymer is a CO_2_-philic chain, and CO_2_-phobic groups should be linked to the side chain of the siloxane polymer molecule through other chemical reactions.^12^

In this study, we present the optimization of the synthetic process for silicone copolymer production using the response surface method (RSM). The structure of the molecule was inferred by Fourier transform infrared (FTIR) spectroscopy, and the thickening ability was measured using a custom-designed falling ball viscometer. The response surface method is used to obtain the optimum operating condition and the most significant interaction parameter among synthetic influencing factors.^17,18^ Our initial attempts in this study mainly focused on synthesizing a new thickener that can easily dissolve in supercritical CO_2_ using toluene as a co-solvent to induce a viscosity increase. A mathematical regression model of the Box–Behnken design was developed to obtain the best reaction conditions and evaluate the interaction among crucial preparation conditions, and the optimum technological condition was verified *via* relevant experiments. A higher yield of the product exists under these synthetic optimal reaction condition. For the viscosity testing, we designed a falling-ball viscometer that could measure the relative viscosity of a mixture of thickener and CO_2_. Finally, we report the trend of the viscosity with the change in the pressure and temperature.

## 2. Experimental

### 2.1 Materials

To prepare the silicone copolymer, numerous silicone compounds with a low molecular weight were obtained from Shanghai Aladdin Bio-Chem Technology Co., Ltd. (China). The remaining chemical reagents were obtained from Nanjing Chemical Reagent Co., Ltd (China). All of the reagents and materials were stored at room temperature and under anhydrous conditions.

### 2.2 Synthesis of silicone copolymer

Octamethylcyclotetrasiloxane (70 g) was mixed with an appropriate amount of tetramethylammonium hydroxide, which was dissolved in 5 ml of water in a three-necked flask containing a N_2_ inlet and outlet. The temperature was slowly increased to the specified temperature, the mixture of the methyl triethoxysilane and aminopropyltriethoxysilane was added to the three-necked flask by a constant pressure dropping funnel, and then the mixture was stirred at the specified temperature under nitrogen gas. After cooling to room temperature, the reaction product was transferred to a separatory funnel, and the aqueous phase was removed through the bottom of the separatory funnel. After equilibration for 2 hours at 25 °C, the volatile chemicals were evaporated at 120 °C for 12 hours under vacuum to yield the target material.

#### FTIR λ

**Fig. 1:**
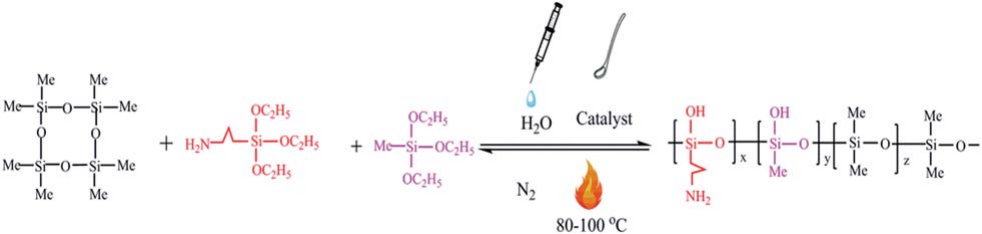
The synthesis process of the silicone ternary copolymer.

**Fig. 2:**
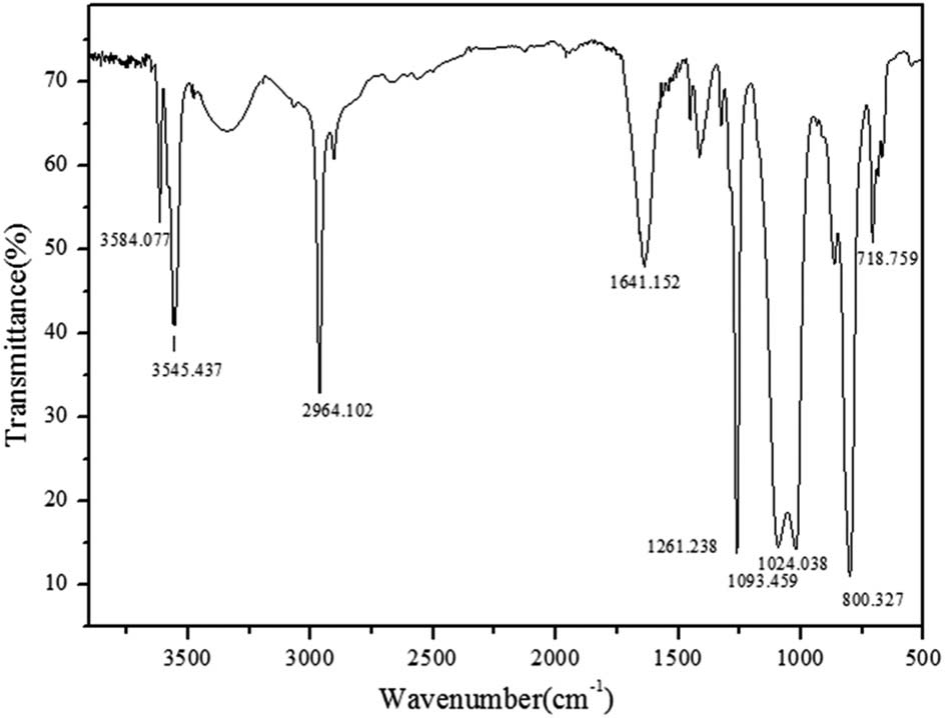
FTIR spectra analysis of silicone copolymer.

The N–H stretching vibrations of the free primary amine were presented by the peaks at 3545 cm^−1^ and 3584 cm^−1^. Moreover, the bending vibration of the peak at 1641 cm^−1^ simultaneously proved the existence of the N–H bond on it. However, the wide band in the range of 3200–3400 cm^−1^ and the peak at 1260 cm^−1^ were considered as the stretching vibration of OH group. The intermolecular association of the polymer indicating that the degree of polymerization was greater than four was attributed to a lower wave number range at 3200–3400 cm^−1^. In addition, the sharp peak at 2964 cm^−1^ was viewed as the C–H stretching vibrations of the CH_3_ connected with Si. Also, the double peak at 1093 cm^−1^ and 1024 cm^−1^ expressed the main chain of Si–O–Si. Similarly, a small peak appeared at 718 cm^−1^, which indicated the swing vibration of the CH_2_–CH_2_. Fig. 2 depicts the analysis of the FTIR spectra for the pure sample. The synthesis process of the silicone copolymer is shown in Fig. 1.

The obtained yields were calculated according to eqn (1):1
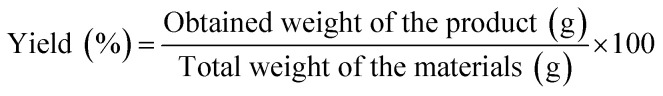


### 2.3 The design of the test equipment for measurement of the thickening ability

**Fig. 3:**
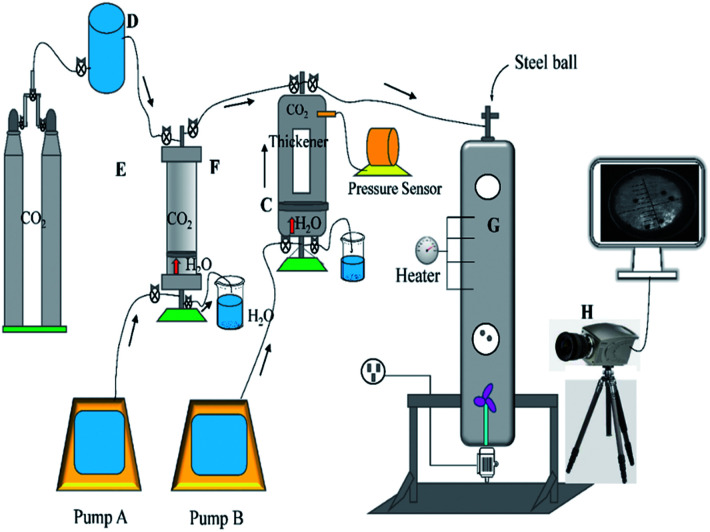
Schematic of the viscosity test system.

Fig. 3 shows a schematic of the viscosity test instrument. It was constructed with four components, which include the injection system, the dissolution system, the viscosity measurement system, and the data processing system. Compressor D was used to pressurize the gas to satisfy the pressure of accumulator F. It should be noted that accumulator F was used to observe the dissolution of the polymer in carbon dioxide under slow pressure. The upper valve 1 of accumulator F was closed when the pressure reached the required value, and the check valve between F and C was set to prevent the mixture in C from returning to F. The CO_2_ in accumulator F was compressed into window regulator C containing thickener by pump A squeezing water to push the piston, and upper valve 1 of C was closed and container C mixed the polymer and CO_2_ by shaking. The pressure of C was detected by a pressure sensor, and pump A pushed the piston to inject CO_2_ into regulator C if the pressure was lower. The solubility of the polymer was clearly observed, and if solid–liquid separation or liquid–liquid separation occurred, that would be considered a failure, and then the thickener and CO_2_ were discharged from the lower valve 2 of C. On the contrary, the mixture of thickener and CO_2_ would be pressed into pressure vessel G, and the sensor was used to detect the pressure and temperature. The blender at the bottom of the container was used to homogenize the liquid, and then the blender was closed and many steel balls were placed into it from the upper valve of G. The valve was then closed, and the falling track was captured by high-speed camera H. At last, the data were sent to the processor.

### 2.4 Experimental model

For the synthetic test of the silicone ternary copolymer, a design was used to screen large influencing factors by response surface methodology in Design Expert 9.0 software. The Box–Behnken design based on the response surface method was employed and optimized into three main reaction conditions, which were aminopropyl triethoxysilane-to-methyl triethoxysilane molar ratio, the weight of the catalyst, and temperature, and the actual values of the 3 factors and the levels that are coded as −1, 0, and +1 are shown in Table 1. Table 2 tabulates the specific experimental yield and the design scheme in BBD. The second-order response surface model was fitted by employing a three-levels-three-factors Box–Behnken design (BBD), and the optimized conditions were predicted by the following second-order polynomial eqn (2):^19^2
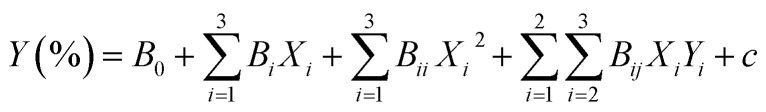
where *Y* represents the yield value; *B*_0_, *B*_*i*_, *B*_*ii*_, and *B*_*ij*_ represent the regression constant, the first-order linear effect, the quadratic (squared) effect, and the cross action of the influencing factors, respectively. The encoded independent variables are expressed as *X*_*i*_, *X*_*j*_, and the random error is represented by *c*.^20,21^

**Table 1:** The experimental range and levels in the Box–Behnken design

Factors	Symbol	Levels		
		−1	0	+1
The amount of catalyst/g	*X*_3_	0.06	0.09	0.12
Molar ratio	*X*_2_	1.5 : 1	2 : 1	2.5 : 1
Temperature/°C	*X*_1_	80	90	100

**Table 2:** Experimental design and the yield of the silicone ternary copolymer for the Box–Behnken design

Run	Temperature/°C	Molar ratio	The amount of catalyst/g	Yield/%
1	0	1	1	76.43
2	0	−1	1	75.17
3	0	0	0	84.01
4	1	1	0	80.37
5	−1	0	1	76.94
6	−1	−1	0	80.31
7	0	0	0	82.13
8	0	0	0	84.51
9	1	0	1	77.01
10	1	−1	0	82.76
11	0	0	0	84.25
12	1	0	−1	74.48
13	−1	1	0	80.32
14	0	0	0	83.71
15	−1	0	−1	77.18
16	0	−1	−1	77.72
17	0	1	−1	76.96

## 3. Results and discussion

### 3.1 ANOVA and the RSM model

The relationship between factors and yields, multiple regression fitting, and the data obtained was estimated by the Box–Behnken design, which relied on the RSM. The multivariate second order equation that was used to investigate the yield of the product after the polymerization reaction is presented as eqn (3):

3 *Y* (%) = 83.72 − 9.5 × 10^−3^ × *A* − 0.24 × *B* − 0.09 × *C* − 0.60 × *A* × *B* + 0.71 × *A* × *C* + 0.51 × *B* × *C* − 1.47 × *A*^2^ − 1.31 × *B*^2^ − 5.84 × *C*^2^

where *Y* denotes the yield of the polymer. *A*, *B*, and *C* indicate the weight of the catalyst (g), the molar ratio of aminopropyl triethoxysilane-to-methyl triethoxysilane, and temperature, respectively.

The data summarized in Table 3 show the analytic evaluations of ANOVA, which fit the second-order response surface model for the yield of the silicone ternary copolymer. The variable is more significant due to the lower *P*-value, and the mathematical model is considered significant when the *P*-value is below 0.05.^19,22^ The high significance level of the fitted model is indicated by the *F*-value of 11.83 and the probability value less than 0.05 in Table 3. The occurrence of the probability of the model *F*-value of 11.83 because of noise is below 0.05. Also, the lack-of-fit *F*-value of 3.01 indicates that it is somehow not significant compared to the pure error. The *P*-value of 0.1577 implied that the model and the data are consistent. Additionally, *R*^2^ was used to evaluate the satisfaction capacity of the model, and in this case, the *R*^2^ value of 0.9383 reveals a small deviation between the actual values and the predicted values. In addition, a high range of the ability and an appropriate goodness-of-fit for the second-order response surface model is conveyed by the correction coefficient of determination (*R*_Adj_^2^ = 0.8590).^25^ For the value of *R*_Pred_^2^ (0.2867), the same conclusion can be made for the adjusted determination coefficient. In addition, a good significant and negligible error is shown by the high value of the predicted determination coefficient. The precision, reliability, and repeatability of the yield of the product are indicated by a small coefficient of variation (CV% = 1.61), and the lower the value, the higher the precision, reliability, and repeatability.^26^

**Table 3:** Analysis of variance for the Box–Behnken design

Source	Sum of squares	DF	Mean square	*F*-value	*P*-value, prob < *F*
Model	174.42	9	19.38	11.83	0.0018
*A*	7.22 × 10^−4^	1	7.22 × 10^−4^	4.41 × 10^−4^	0.9838
*B*	0.44	1	0.44	0.27	0.6188
*C*	0.068	1	0.068	0.042	0.8438
*AB*	1.44	1	1.44	0.88	0.3789
*AC*	1.99	1	1.99	1.21	0.3070
*BC*	1.02	1	1.02	0.62	0.4559
*A* ^2^	9.09	1	9.09	5.55	0.0507
*B* ^2^	7.27	1	7.27	4.44	0.0731
*C* ^2^	143.60	1	143.60	87.67	<0.0001*
Residual	11.47	7	1.64		
Lack of fit	7.94	3	2.65	3.01	0.1577
Pure error	3.52	4	0.88		
Cor total	185.89	16			

As shown in Table 3, terms are indicated as significant if the *P*-value is below 0.0001.^23,24^*C*^2^ is considered as significant by ANOVA because of the smaller *P*-value in Table 3. The *P*-value of *C* has an adequate influence on the yield of the product because the *P*-value is less than 0.0001. On the contrary, the numerical value shows that *A*, *B*, *C*, *AB*, *AC*, *BC*, *A*^2^, and *B*^2^ have no significant effect. These terms could be deleted and a modified model of this synthetic process could be generated.

The reason for the workings of the above model is summarized as follows. A large amount of energy was absorbed when the bond between the Si atom attacked by the catalyst and the O atom adjacent to this Si broke, and the bond of Si–O was easy to break with increasing reaction temperature (80–100 °C). Moreover, as an endothermic reaction, the hydrolysis reaction of methyl triethoxysilane and aminopropyltriethoxysilane requires the absorption of a great deal of energy, and the above three reactions showed a more sufficient reaction efficiency and a higher yield with the rise in temperature. Additionally, the molar ratio of methyl triethoxysilane and aminopropyltriethoxysilane and the amount of catalyst exhibited a smaller effect on the yield of the product. For the molar ratio, a decreased addition of methyl triethoxysilane and aminopropyltriethoxysilane were insignificant factors. For the amount of catalyst, the increase in yield of synthetic polymer was very small when the catalyst reached a certain amount. The catalyst affects only the reaction rate of the polymerization reaction, and there was a larger collision probability between the catalyst molecule and the octamethylcyclotetrasiloxane molecule to cause the shorter reaction time, but the smaller change for the yield of product is shown in this study.

**Fig. 4:**
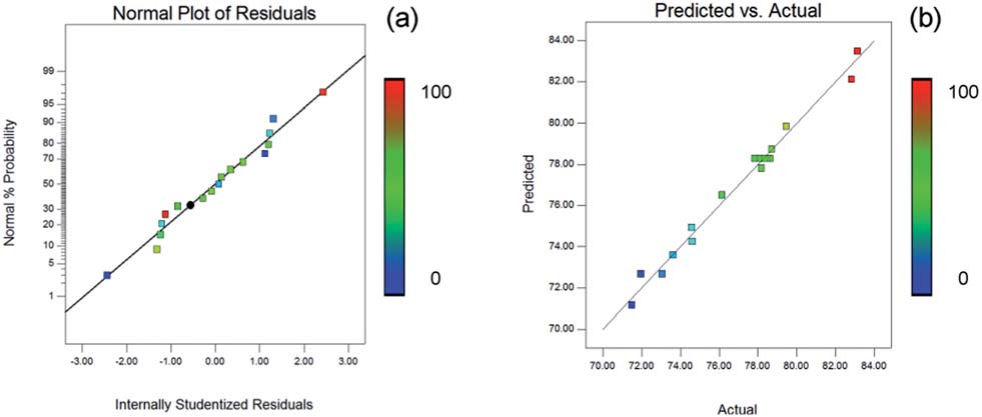
The adequacy and the normality assumption of the model. The color change from blue to red represents a gradual increase in the yield of the product.

The residuals could be used to evaluate the adequacy of the model and the normality assumption. The normal probability plot of the residuals is shown in Fig. 4a. The data points were uniformly distributed around a straight line, which was similar to the plots of normal probability *versus* the standardized residual. Based on this line, the independence of the residuals was clearly confirmed. As illustrated in Fig. 4b, it is observed that there are similar trends of the plot of the actual *versus* predicted values being close to a straight line. The model was considered accurate according to the diagonal line along which these points cluster in Fig. 4.

### 3.2 Analysis of single factors

**Fig. 5:**
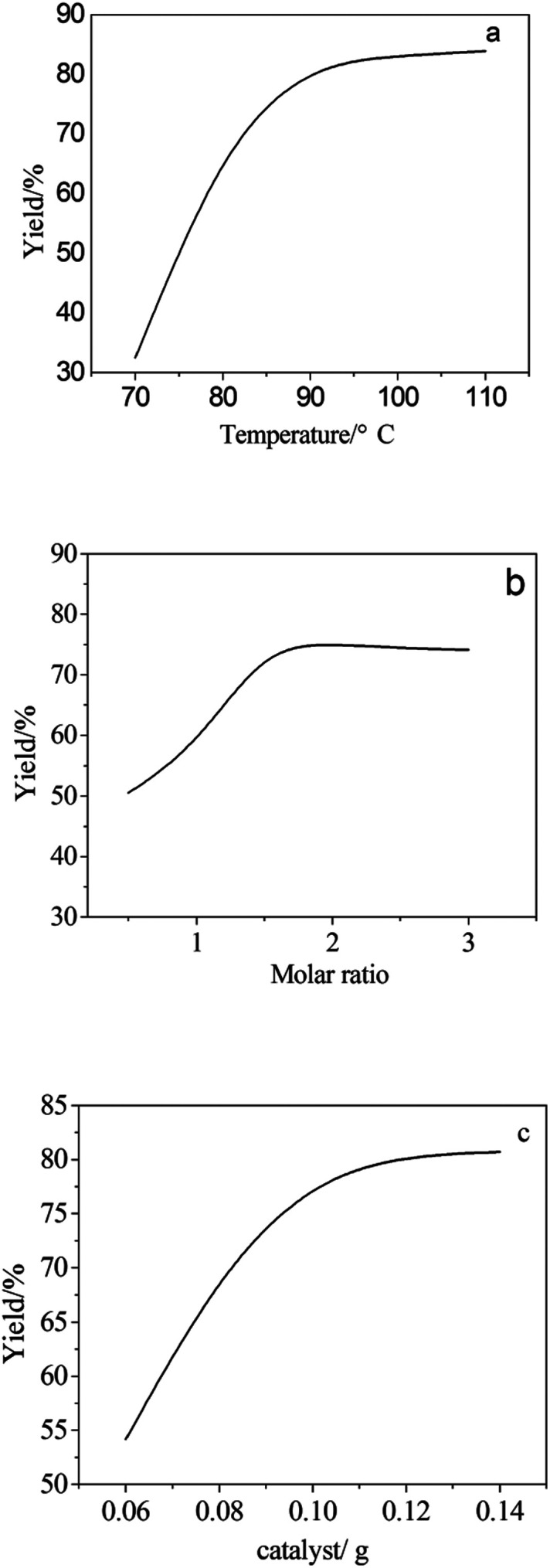
Line chart of a single factor with the yield. (a) Temperature, (b) molar ratio, and (c) the amount of catalyst.

The relation between temperature and yield is shown in Fig. 5a. With a molar ratio of 2 : 1 and a catalyst amount of 0.09 g, the higher the temperature, the greater the yield. Additionally, the yield did not increase until the temperature was higher than 92 °C. The optimum synthesis temperature along the linear trend was 90 °C. Fig. 5b shows a similar phenomenon, where the relation between the aminopropyl triethoxysilane-to-methyl triethoxysilane molar ratio and the yield is a positive correlation when the temperature is 90 °C and the molar ratio is 2 : 1. Then, it can be concluded that the most suitable molar ratio is 2 : 1, due to synthetic efficiency and cost. In addition, the direct proportion is shown in Fig. 5c when the temperature and molar ratio are under certain circumstances. A similar trend appears in Fig. 5b and c, where an incremental yield changes along with the change of a single factor. The best conditions were considered to be a temperature of 90 °C, molar ratio of 2.5 : 1, and catalyst amount of 0.09 g only when the other two single factors remained unchanged.

### 3.3 The reciprocal effect between various factors

**Fig. 6:**
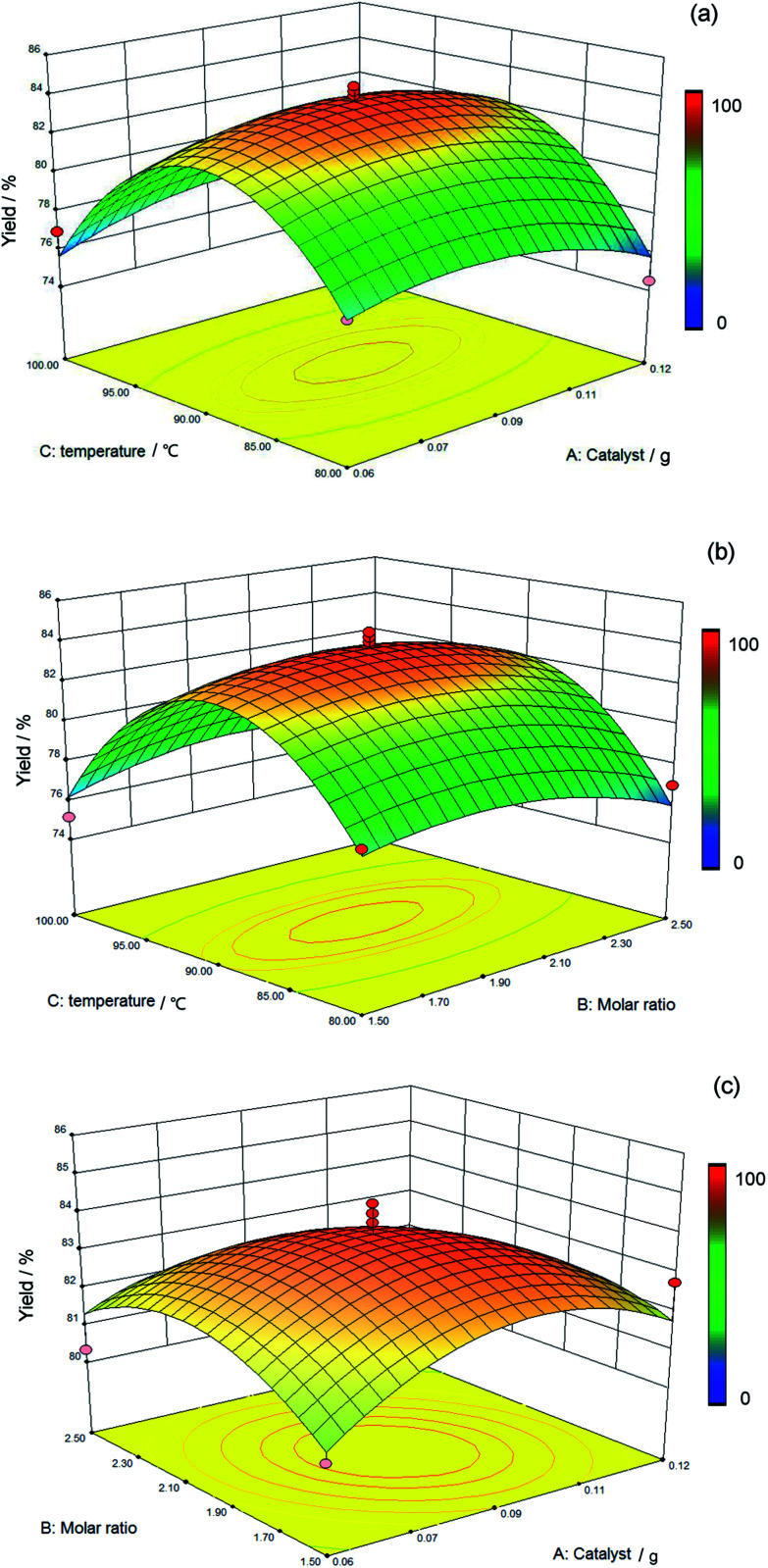
Response surface plots showing the effect of every parameter on yield. The color change from blue to red represents a gradual increase of yield of the product. (a) The interaction of temperature and catalyst. (b) The interaction of temperature and molar ratio. (c) The interaction of catalyst and molar ratio.

Although the impact of each factor on the results was indicated on the condition that other factors remain constant in Fig. 5, the effect of a single factor on the yield was not representative of the experiment. Based on this situation, the interaction of different factors *via* response surfaces and contour plots was investigated in this study. A more intuitive visualization of the mutual effect of the system response between two random influencing factors such as temperature, molar ratio, and weight of catalyst is displayed in Fig. 6.

The influence of the interaction of temperature and catalyst upon the yield of the silicone ternary copolymer was determined using the three-dimensional response surface and two-dimensional contour plots, as shown in Fig. 6a. The molar ratio of 2 : 1 and reaction time of 5 h are seen as the central point, and it was also considered that the reaction of two factors was prominent because of the steep contour (line). The yield of the silicone ternary copolymer was observed to increase with increasing reaction temperature (80–90 °C). The highest yield of 83.02% was obtained at 90 °C. However, with higher temperatures (>90 °C), there was a decreasing trend of the yield. This occurred because the hydrolysis reaction was obstructed due to the high temperature. The yield also decreased with increasing temperature due to the volatilization of methyl triethoxysilane, aminopropyltriethoxysilane, and water. The yield did not change based on the amount of catalyst in the range of 0.06 g to 0.12 g, and sufficient catalyst was the primary reason.

Fig. 6b describes the effect of temperature as well as the molar ratio and the interaction of the above two factors on the yield value, and the amount of catalyst of 0.09 g was a prerequisite. The same regular pattern is presented in Fig. 6b that is comparable with Fig. 6a. More specifically, the change in temperature presents a greater influence on the yield than the molar ratio of the reagents.

Based on Fig. 6c, the interaction of the weight of the catalyst and the molar ratio was more insignificant compared with the other two interactions that are shown in Fig. 6a and b, and the central point was defined as the reaction temperature of 90 °C at a constant time of 5 h. Fig. 6c depicts a maximum value of the yield of silicone ternary copolymer at a temperature of 90 °C, a catalyst amount of 0.1 g, and a molar ratio of 2 : 1. The stable value of the yield of the product was found to be suitable for the range of the catalyst levels (0.06–0.12 g) and the molar ratio (1.5 : 1–2.5 : 1). More specifically, the temperature was the predominant factor that increased the yield, and the catalyst and molar ratio were in a reasonable range at this time.

Fig. 6a–c shows the interaction of temperature with the reactant ratio and the interaction of temperature with a catalyst for the yield of copolymer, and a significant influence is clearly presented. On the contrary, the smallest factor is shown by the interaction between the molar ratio and the catalyst. Overall, the greatest effect on polymer yield among the three factors investigated was caused by temperature.

### 3.4 Analysis of the viscosity equation

**Fig. 7:**
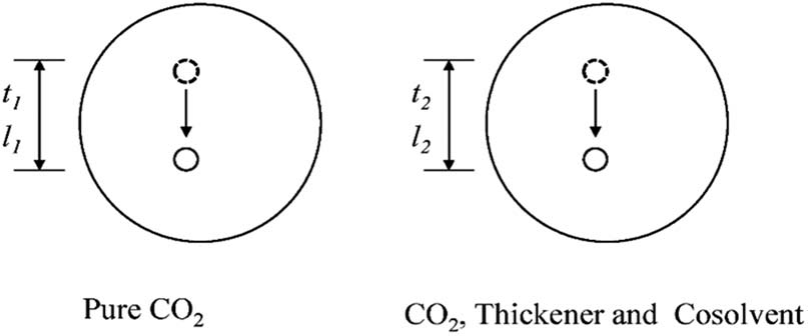
Schematic of the viscosity calculation for the silicone thickener.

The custom-designed measurement equipment is shown in Fig. 3. The basic principle and schematic of the viscosity calculations for the silicone thickener are presented in Fig. 7.

A schematic showing the viscosity calculation for the silicone thickener is presented in Fig. 3. For a falling ball viscometer, accurate viscosity could not be obtained for the silicone thickener, but the multiples of viscosity between the mixture (silicone thickener and CO_2_) and pure CO_2_ were calculated precisely as shown in eqn (4), and it was designated as the relative viscosity. Compared with the capillary viscometer, the position of the steel ball at a specific time in the fluid was captured, and the average speed (*v*_1_ or *v*_2_) was solved according to the time difference (*t*_1_ or *t*_2_) and distance (*l*_1_ or *l*_2_). Obviously, the steel ball should maintain a state of equal velocity motion in the liquid, and the speed required (*v*_1_ or *v*_2_) should be considered as the average value of the velocity of three different distances in the uniform motion stage:4
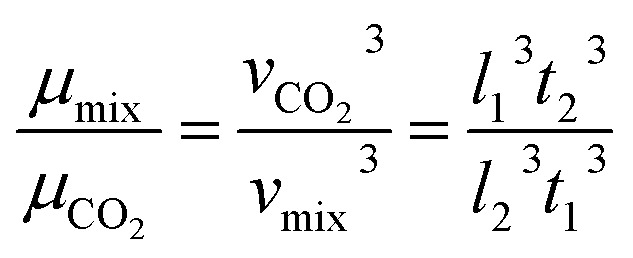
where *η*_CO_2__ and *η*_mix_ are the viscosity of the pure CO_2_ and the mixture, respectively, and *v*_1_ presents the speed of the steel ball for the stable distance (*l*_1_) and the stable time (*t*_1_) in pure CO_2_. Similarly, *v*_2_ expresses the speed of the steel ball for the stable distance (*l*_2_) and the stable time (*t*_2_) in a mixture of silicone thickener and CO_2_.

Measurement equipment was custom-designed based on Stoke's law,^27^ and eqn (4) provides an analytical expression for the viscosity. In the state of uniform motion, Stoke's law and eqn (4) are applied. The two forces denoted as eqn (5) and (6) are always present during the descent of the ball.^28^

5 *mg* = *V*_0_*ρ*_s_*g* = *G*

6
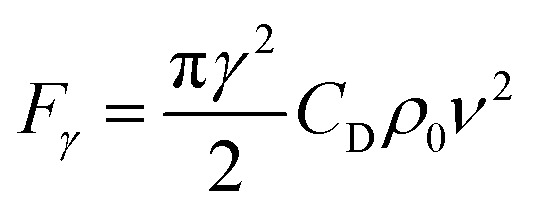

7 *F* = *V*_0_*ρ*_0_*g*

The steel ball presents the state of force balance when moving at a constant speed. Hence, equating these three (eqn (5)–(7)), the force on the ball can be described as follows:

8 *mg* − *F*_r_ − *F* = 0

9
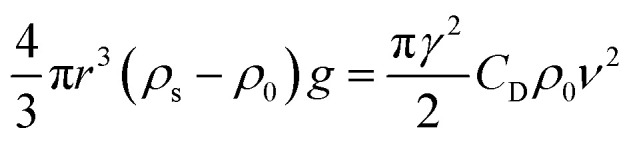
10
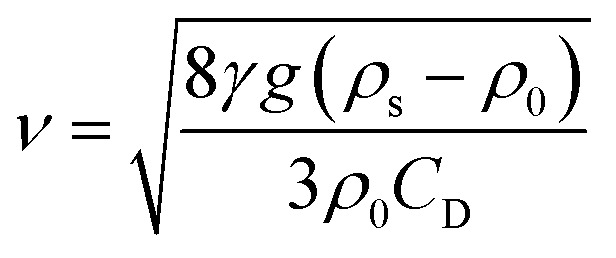
where *ν* is the ball velocity at the state of a constant speed in the fluid, *C*_D_ presents the drag coefficient of the fluid, *γ* denotes the ball radius, *ρ*_s_ is the density of the ball, *ρ*_0_ is the density of the fluid, *F*_r_ is the resistance to the ball, and buoyancy is marked with *F*. Eqn (9) shows that the falling velocity of the ball is maintained at a constant state when gravity and resistance are equal. Overall, the constant speed of the ball decreases with the increase in the viscosity of the fluid.11
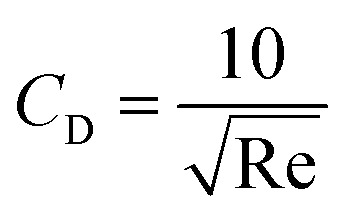
12
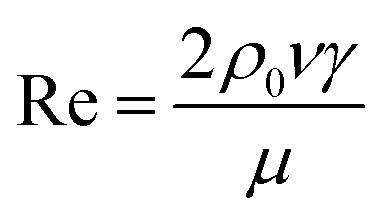
13
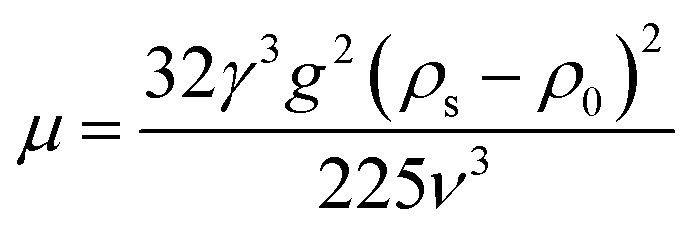


As a supercritical fluid, Re = 23.03 was measured for CO_2_. Similarly, Re = 0.43 was obtained when 6.4 wt% siloxane was added to pure CO_2_. Eqn (11), which was used for Re in the range of 0.1 to 10^3^, satisfied the requirement at the range of 0.43 to 24. Supercritical CO_2_ showed a laminar state due to the Re. Moreover, there was almost no change in the density of the fluid after a small amount of thickener and co-solvent were added.^14^ The laminar state still existed according to Re = 0.43. The relative viscosity between the mixture fluid and pure CO_2_ could simultaneously be considered as eqn (14).14
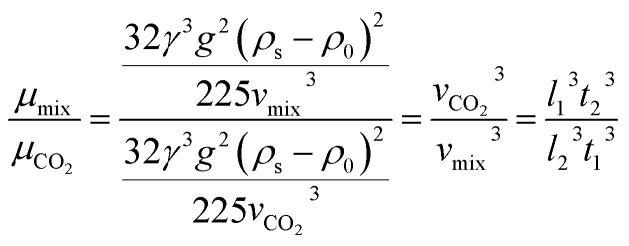


Compared with other measurement equipment, an accurate viscosity could not be obtained according to eqn (14), and the simplicity of the viscosity calculations is shown.

### 3.5 Measurement of the thickening ability of the silicone polymer

Table 4 presents the data for the relative viscosity of the different concentrations of thickener under different measurement conditions. As can be seen in Table 4, the viscosity increases with the increased concentration of polymer under the same conditions measured.^12^ On the contrary, a reduced trend is shown in Table 4 at the same concentration temperature and when the pressure increased. The effect of pressure on solubility of the polymer was a major cause of the tendency for a decrease in the viscosity of the fluid.^29–31^ In addition, a downward trend is presented in Table 4 that occurred when the temperature changed. A decrease in viscosity also occurred because the solubility of polymer decreased with the decreasing temperature.^32,33^ More specifically, the solubility was an important prerequisite for thickening properties, and the density of CO_2_ that was associated with temperature and pressure affected the solubility of compounds. Indirectly speaking, temperature and pressure restricted the thickening performance.

**Table 4:** Measurement conditions and relative viscosity of the mixture fluid (with toluene as a cosolvent)

Polymer (wt%)	Cosolvent (wt%)	Temp (°C)	Pressure (MPa)	Relative viscosity
1	7	35	8	1.8
1	7	45	8	1.4
1	7	55	8	1.2
1	7	35	10	2.3
1	7	45	10	1.8
1	7	55	10	1.7
1	7	35	12	2.4
1	7	45	12	2.3
1	7	55	12	1.8
3	7	35	8	5.1
3	7	45	8	4.6
3	7	55	8	4.1
3	7	35	10	5.5
3	7	45	10	4.8
3	7	55	10	4.1
3	7	35	12	5.7
3	7	45	12	4.9
3	7	55	12	4.2

**Fig. 8:**
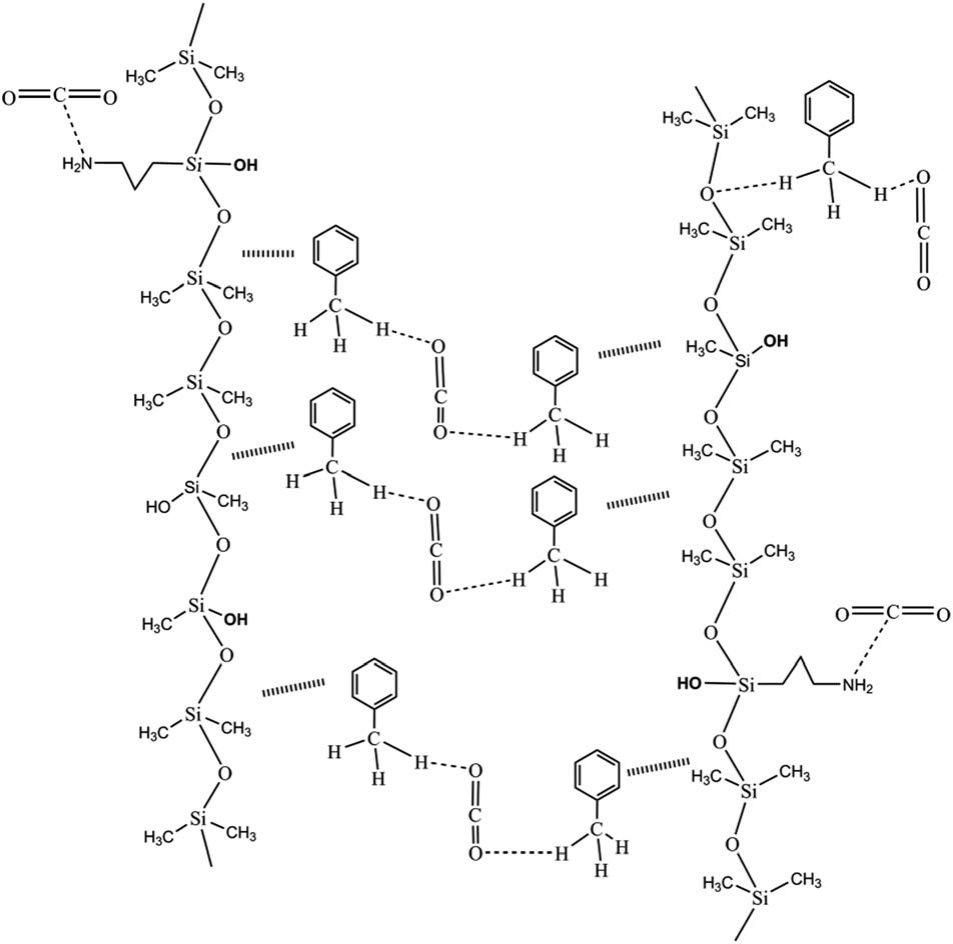
The mechanism used by the silicone polymer to thicken CO_2_.

The thickening properties were the result of the combination of the following groups. As an electronic group, the NH_2_ in siloxane interacted with the CO_2_. More specifically, the lone pair of N that interacts with C lacked electrons in CO_2_, and CO_2_ was above the N.^34–36^ The OH in siloxane played a role in increasing the space grid structure formed by the polymer and CO_2_ molecules. In addition, the O of CO_2_ interacted with the C–H of the methyl group in toluene. Similarly, the C–H⋯O bond^43–45^ between the O of the siloxane backbone and the C–H of the methyl group in toluene explained the increased CO_2_ viscosity of the silicone-containing polymer. The thickening mechanism is shown in Fig. 8. A suitable ratio of CO_2_-phobic segments not only affected the solubility with the assistance of toluene but also improved the thickening performance.^42^ More specifically, a certain number of OH moieties improved the space grid structure to thicken the CO_2_. Nonetheless, the small intermolecular forces that result from big chain flexibility are the important factors that thickened the CO_2_.

### 3.6 Environmental assessment

Fluoropolymers have been widely researched in the field of CO_2_ thickeners because of their increased ability to solubilize.^37–39^ However, environmental pollution is the biggest obstacle to applying this compound.^40^ In contrast, siloxane polymer is advantageous because it is inexpensive and environmentally friendly, and it subsequently has attracted the interest of researchers around the world.^12–14^ Moreover, comparisons between the water consumption of hydraulic fracturing with that of CO_2_ fracturing technology have revealed some significant differences during oilfield development, and water pollution was increased. Furthermore, greenhouse gas emissions have also decreased based on CO_2_ fracturing technology.

## 4. Conclusion

In this study, silicone copolymer was successfully produced *via* a one-step synthesis as compared to previously reported studies that required a multi-step synthesis route. Design-Expert software was used to optimize the polymerization process of this polymer. From the statistical analyses, the empirical second-order polynomial can accurately describe this model, and the experimental data are consistent with the predicted value. The optimum process parameters were determined as follows: temperature 90 °C, an aminopropyltriethoxysilane-to-methyl triethoxysilane molar ratio of 2 : 1, and the amount of tetramethylammonium hydroxide 0.09 g. Under these conditions, the resulting polymer yield was 83.72% with an accuracy of 93.83%.

The measurement equipment included a custom-designed falling-ball viscometer that was utilized to measure the thickening properties. The results showed that the relative viscosity of CO_2_ increased 5.7 fold upon addition of the mixed silicone copolymer fluid, which was inexpensive and more environmentally friendly than the polymers that have been described in previously published studies.

Water resources can be effectively protected by using a silicone polymer rather than a fluoropolymer. More importantly, CO_2_ fracturing technology could actualize the storage of CO_2_ to significantly ameliorate the greenhouse effect.

## Conflicts of interest

There are no conflicts to declare.

## Supplementary Material
